# Transapical Aortic Valve Implantation in Bicuspid Aortic Valves: Must Be an Absolute Contraindication?

**DOI:** 10.5812/cardiovascmed.4498

**Published:** 2012-11-01

**Authors:** Emiliano A. Rodríguez-Caulo, Omar A. Araji, José M. Barquero

**Affiliations:** 1 Cardiovascular Surgery Department, Virgen Macarena Universitary Hospital, Sevilla, Spain

**Keywords:** Heart Valve Prosthesis Implantation, TAVI, Endovascular Procedures, Aortic Valve

## Abstract

Transcatheter Aortic Valve Implantation (TAVI) is a new therapeutic option for patients with severe aortic stenosis with unacceptable surgical risk for conventional aortic valve surgery. A Bicuspid Aortic Valve (BAV) is the most common congenital cardiac disorder (1% of the population) and currently is considered exclusion criteria for TAVI, because it predicts an increased risk of adverse aortic events as incomplete sealing, severe paravalvular regurgitation, or dislocation due to more frequent elliptic shape and asymmetric calcifications in BAV annulus.

Only few cases have been published in recent literature, so in this case report we illustrate our experience and management of TAVI in a BAV, with excellent outcomes and no late complications at 1 year follow-up.

We believe that currently the presence of a BAV might not be considered an absolute contraindication for TAVI, because although there is no sufficient data for assess the safety or efficacy of TAVI in BAV, this case report shows that it could be performed safely in selected patients with unacceptable surgical risk after an extensive preoperative evaluation, avoiding this procedure in patients with bad prognostic factors as huge and heavy calcifications, asymmetric valves, elliptic annulus or small distance from leaflets to coronary ostia. Each case must be individualized, being alert at follow-up because the risk of late complications.

## 1. Introduction

Transcatheter Aortic Valve Implantation (TAVI) is an emerging therapeutic option for patients with severe aortic stenosis (AS) who are considered at high risk for conventional aortic valve surgery (1). Bicuspid Aortic Valves (BAV) occurs in approximately 1% of the population, being the most common congenital cardiac disorder. Initially the presence of a BAV was considered exclusion criteria for TAVI, because it predicts an increased risk of adverse aortic events, as progressive aortic annulus dilation with secondary device dislocation, or malfunctioning (2). Only few cases have been published in recent literature. In this case report we illustrate our experience with TAVI in a BAV.

## 2. Case Report

We present a 75-year-old female with symptomatic AS, with calcified BAV, hypertensive, diabetic, with severe pulmonary hypertension (systolic pulmonary artery pressure of 62 mmHg) and chronic obstructive pulmonary disease, presenting a logistic Euroscore I of 21.9%. Due to the high risk for conventional aortic valve replacement, a TAVI transapical approach was decided as the best option due to very small and calcified femoral arteries (6 mm). We explained to the patient that BAV was currently considered an absolute TAVI contraindication, but emphasizing that due to the morphology of the valve, the TAVI procedure was feasible and likely to be less invasive and risky. He accepted the risk of the procedure and signed informed consent.

**Figure 1.**
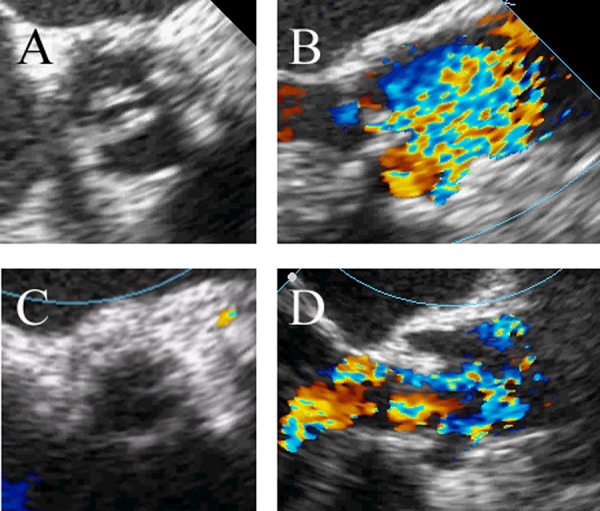
Intraoperative images from TEE. Preoperative BAV short (A) and long axis (B) versus postoperative short (C) and long axis (D) after Edwards Sapien valve deployment, without evidence of malpositioning nor paravalvular leak.

**Figure 2.**
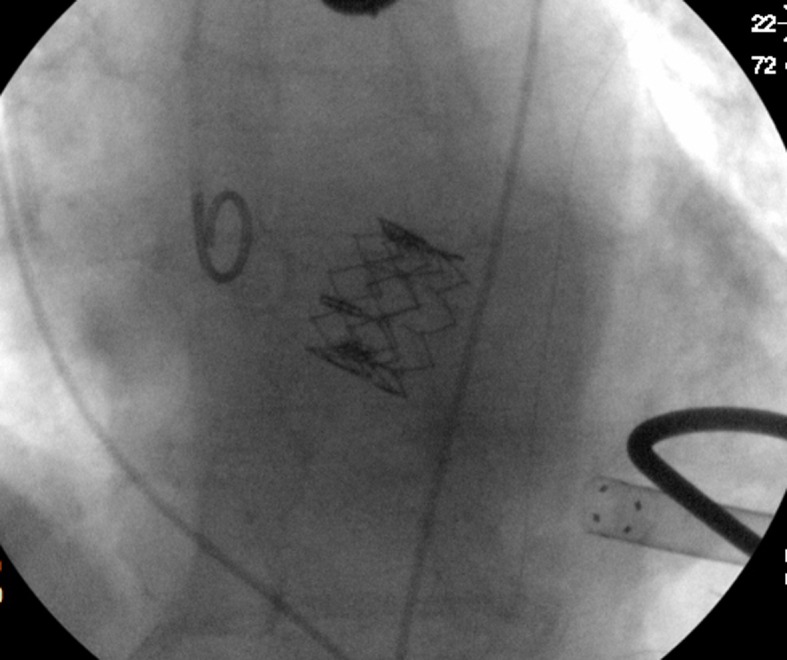
C-arm fluoroscopy image after Edwards Sapien homogeneous deployment.

Intraoperative transesophageal echocardiography (TEE) confirmed a mean aortic gradient of 55 mmHg with a surface area of 0.8 cm2 and a 19 mm not too elliptic aortic annulus. Under general anaesthesia, transapical TAVI was performed uneventfully under fluoroscopic and TEE guidance. After the standard TAVI protocol (2), a 23 mm Edwards Sapien valve (Edwards Lifesciences Inc, Irvine, CA) was successfully implanted without misplacement nor paravalvular leak, and with complete circular valve expansion confirmed by intraoperative TEE and fluoroscopy (Figures 1 and 2). The patient was discharged without complications on 12th postoperative day.

## 3. Discussion

Currently, the presence of a BAV is a contraindication for TAVI regardless the selected approach, although BAV is the most common cardiac disorder, comprising 3-5% of patients undergoing aortic valve replacement (3). This is because it exists an increased probability of incomplete sealing, severe paravalvular regurgitation, dislocation or malpositioning due to more frequent elliptic shape and asymmetric calcifications in BAV annulus (2).

Nowadays there is little clinical experience and evidence regarding this issue, with only few reports in literature (3, 4). In the largest series of 11 BAV TAVI procedures (4), circular valve expansion was achieved in almost all cases, demonstrated by intraoperative TEE, showing that BAV does not preclude an effective sealing. Nevertheless the demonstrated feasibility of the procedure, a high rate of complications were described (6/11, 55%), with 2 early deaths, 3 early moderate paravalvular leaks, and one late conversion to open surgery due to valve migration, so it is a very important issue to consider.

Although there is no sufficient data to ensure the safety or efficacy of TAVI in BAV, our study shows it could be done safely in selected patients with unacceptable surgical risk, if a comprehensive preoperative evaluation of the patient´s morphology valve was performed (computed tomography, TEE, 3D echocardiography), avoiding this procedure in BAV with extreme calcifications, extensive asymmetric valves, very elliptic annulus or small distance from leaflets to the coronary ostia. Oversizing the valve 15-20% over his minor elliptic BAV diameter is needed to achieve a correct anchorage and prevent displacement and migration (4).

The success rate depends on patient selection, so it is very important to exclude those with high risk for device failure. Further studies to confirm long-term durability of the device are required, with follow-up echocardiography and multi-slice computed tomography to assess function and correct positioning of the valve in BAV annulus (5).

At 12 months, no late complications were reported.

## 4. Conclusions

Currently, the presence of a BAV might not be considered as an absolute contraindication for TAVI, because it can be performed safely after a thorough preoperative evaluation. Each case must be individualized, being alert because the risk of late complications. Further follow-up and studies are needed to reveal exact contraindications for TAVI.
